# Concerted and differential actions of two enzymatic domains underlie Rad5 contributions to DNA damage tolerance

**DOI:** 10.1093/nar/gkv004

**Published:** 2015-02-17

**Authors:** Koyi Choi, Sabrina Batke, Barnabas Szakal, Jonathan Lowther, Fanfan Hao, Prabha Sarangi, Dana Branzei, Helle D. Ulrich, Xiaolan Zhao

**Affiliations:** 1 Molecular Biology Program, Memorial Sloan Kettering Cancer Center, New York, NY 10065, USA; 2 Programs in Biochemistry, Cell, and Molecular Biology, Weill Graduate School of Medical Sciences of Cornell University, New York, NY 10021, USA; 3 Institute of Molecular Biology, Ackermannweg 4, 55128 Mainz, Germany; 4 IFOM, Fondazione Istituto FIRC di Oncologia Molecolare, Milan, Via Adamello 16, 20139, Italy; 5 Cancer Research UK London Research Institute, Clare Hall Laboratories, Blanche Lane, South Mimms, EN6 3LD, UK

## Abstract

Many genome maintenance factors have multiple enzymatic activities. In most cases, how their distinct activities functionally relate with each other is unclear. Here we examined the conserved budding yeast Rad5 protein that has both ubiquitin ligase and DNA helicase activities. The Rad5 ubiquitin ligase activity mediates PCNA poly-ubiquitination and subsequently recombination-based DNA lesion tolerance. Interestingly, the ligase domain is embedded in a larger helicase domain comprising seven consensus motifs. How features of the helicase domain influence ligase function is controversial. To clarify this issue, we use genetic, 2D gel and biochemical analyses and show that a Rad5 helicase motif important for ATP binding is also required for PCNA poly-ubiquitination and recombination-based lesion tolerance. We determine that this requirement is due to a previously unrecognized contribution of the motif to the PCNA and ubiquitination enzyme interaction, and not due to its canonical role in supporting helicase activity. We further show that Rad5′s helicase-mediated contribution to replication stress survival is separable from recombination. These findings delineate how two Rad5 enzymatic domains concertedly influence PCNA modification, and unveil their discrete contributions to stress tolerance.

## INTRODUCTION

Faithful DNA replication is essential for the maintenance of genome integrity. Various mechanisms can facilitate this process under genome stress situations. Among them, lesion tolerance (also called DNA damage tolerance or post-replicative repair) uses both recombination- and translesion synthesis-based mechanisms to facilitate damage bypass. In eukaryotes, lesion tolerance is largely controlled by the ubiquitination of proliferating cell nuclear antigen (PCNA) (reviewed in 1-3). Using budding yeast as an example, when PCNA is mono-ubiquitinated by the ubiquitin E2 and E3 (or ligase) pair, Rad6 and Rad18, damage-tolerant polymerases are recruited to initiate translesion synthesis. Extending this modification to poly-ubiquitination by another E2 and E3 pair, the Mms2-Ubc13 dimer and Rad5, enables recombination-mediated mechanisms. In this route, Rad51 and other recombination factors generate joint DNA molecules allowing DNA to be synthesized from the sister strand. These joint molecules are eventually resolved by resolution or dissolution enzymes with the help of specific regulators, such as the conserved Smc5/6 complex (4-7). Both branches of the lesion tolerance pathway contribute to replication stress tolerance and genome stability, and have direct implications in human diseases, particularly cancer and cancer-prone syndromes (8-12).

While most enzymes in this pathway exhibit a single activity, Rad5 has several. Most relevant here, it catalyzes PCNA poly-ubiquitination and exhibits DNA-dependent adenosine triphosphatase (ATPase) activity (13-17). In its first function, Rad5 bridges PCNA with the E2 (Mms2-Ubc13) and accelerates ubiquitin transfer from the E2 to PCNA (14,17). As a DNA-dependent ATPase, Rad5 is a member of the DEAD box family of helicases, and catalyzes the reversal of replication fork-like structures *in vitro* (8,13,16). Replication fork reversal in principle can lead to recombination-based lesion bypass (reviewed in 18,^19^), raising the possibility that the Rad5 helicase function collaborates with its ubiquitin ligase activity during recombination-mediated processes. Incidentally, the relevant catalytic domains of Rad5 overlap. The ligase domain (a RING E3 domain) responsible for E2 interaction resides within the helicase domain, inserted between the conserved helicase motifs III and IV (8,20-22).

The multiple activities and overlapping domains seen for Rad5 are conserved among its homologs, including the human tumor suppressors SHPRH and HLTF (reviewed in 2,23). Thus, these shared features may be of physiological importance. However, a consensus regarding how the different, yet overlapping, activity domains functionally relate to each other has not been reached. Both dependent and independent relationships between the two domains of Rad5 have been proposed. Mutations of individual Rad5 helicase motifs show either epistatic or additive genetic relationships with mutations affecting PCNA poly-ubiquitination (9,11,24,25). These results imply very different models for how Rad5 functions in damage tolerance. For example, Chen *et al*. suggest that Rad5 helicase function is independent of its ubiquitin ligase function (25). This was challenged by a recent study that indicates that the two work sequentially and not independently (11), whereas another recent study suggests that it could be either (24).

To better understand how the different activities of Rad5 relate to each other and how they contribute to replication stress tolerance, we examined *rad5* mutations at two highly conserved helicase motifs in a battery of tests. Our results show that an intact Walker B motif essential for ATP interaction is required for PCNA poly-ubiquitination, consistent with a similar finding by Ball *et al*. (24). Our mechanistic studies further suggest that this requirement is not due to the canonical role of this motif to support ATP hydrolysis, but rather an additional role in facilitating substrate-enzyme interaction. Using a second helicase motif mutation, we show that Rad5 ATPase activity *per se* is not required for PCNA poly-ubiquitination and contributes to lesion tolerance independently of recombination. These results reveal a new role for the Rad5 helicase domain in ubiquitination through supporting substrate-enzyme interaction and delineate both concerted and differential effects of the two Rad5 functional domains, thus reconciling different models of Rad5 function.

## MATERIALS AND METHODS

### Yeast strains, plasmids and yeast manipulation

The yeast strains and two-hybrid plasmids used in this study are listed in Table 1. Strains are derivatives of W1588–4C, a *RAD5* derivative of W303 (*MATa ade2–1 can1–100 ura3–1 his3–11,15 leu2–3,112 trp1–1 rad5–535*) (26). Only one strain for each genotype is listed, but at least two independent spore clones of each genotype were used in each of the experiments. Standard procedures were used for strain construction, growth and medium preparation.

**Table 1.** Yeast strains and plasmids used in this work

Name	Genotype	Sources
W1588–4C	*MAT*a *ade2–1 can1–100 his3–11,15 leu2–3,112 trp1–1 ura3–1 RAD5+*	R. Rothstein
Z361	*MATα rad5-AA*	this study
T638	*MATα rad5Δ::KAN*	lab collection
T585	*MAT*a *RAD5-TAP::HIS3*	lab collection
T382-P4	*MAT*a *smc6-P4–13MYC::HIS3*	(5)
T605–14B	*MAT*a *smc6-P4–13MYC::KAN*	(30)
X3243–3D	*MAT*a *rad5-AA smc6-P4–13MYC::KAN*	this study
X1266–2C	*MAT*a *smc6–56–13MYC::HIS3*	(5)
T606–7A	*MAT*a *smc6–56–13MYC::KAN*	(30)
X3244–13C	*MAT*a *rad5-AA smc6–56–13MYC::KAN*	this study
W3111–1C	*MAT*a *rad51Δ::LEU2*	R. Rothstein
X3307–2C	*MAT*a *rad5-AA rad51Δ::LEU2*	this study
X3307–2B	*MATα rad51Δ::LEU2 smc6-P4–13MYC::KAN*	(5)
X3307–3A	*MAT*a *rad51Δ::LEU2 rad5-AA smc6-P4–13MYC::KAN*	this study
X3311–2C	*MATα rad51Δ::LEU2 smc6–56–13MYC::KAN*	(5)
X3311–3A	*MAT*a *rad51Δ::LEU2 rad5-AA smc6–56–13MYC::KAN*	this study
T645	*MAT*a *mph1Δ::URA3*	(5)
X3312–3D	*MAT*a *mph1Δ::URA3 smc6–56–13MYC::KAN*	(5)
X3312–6A	*MAT*a *mph1Δ::URA3 rad5-AA smc6–56–13MYC::KAN*	this study
W2889–19B	*MAT*a *shu1Δ::HIS3*	R. Rothstein
X3313–13A	*MAT*a *shu1Δ::HIS3 smc6–56–13MYC::KAN*	(30)
X3313–14B	*MAT*a *shu1Δ::HIS3 rad5-AA smc6–56–13MYC::KAN*	this study
T767	*MAT*a *mms2Δ::HIS3*	(30)
X3314–7C	*MATα mms2Δ::HIS3 smc6–56–13MYC::KAN*	(30)
X3314–3D	*MATα mms2Δ::HIS3 rad5-AA smc6–56–13MYC::KAN*	this study
T770	*MAT*a *esc2Δ::KAN*	(30)
X3245–1C	*MAT*a *esc2Δ::KAN rad5-AA*	this study
W1958–4D	*MATα sgs1Δ::HIS3*	R. Rothstein
X3246–13D	*MATα sgs1Δ::HIS3 rad5-AA*	this study
X3826–8D	*MAT*a *pol30::URA3 leu2::YIp128-HisPOL30[LEU2]*	this study
X3856–12C	*siz1Δ::KAN pol30::URA3 leu2::YIp128-HisPOL30[LEU2]*	this study
X3857–1B	*rad18Δ::LEU2 pol30::URA3 leu2::YIp128-HisPOL30[LEU2]*	this study
X3825–4B	*mms2Δ::KAN pol30::URA3 leu2::Yip128-HisPOL30[LEU2]*	this study
X3824–1A	*rad5Δ::KAN pol30::URA3 leu2::Yip128-HisPOL30[LEU2]*	this study
X3823–6C	*MATα rad5-AA pol30::URA3 leu2::YIp128-HisPOL30[LEU2]*	this study
X5391–1–4B	*smc6–56–13MYC::KAN pol30::URA3 leu2::YIp128-HisPOL30[LEU2]*	this study
X5391–1–1C	*rad5-AA smc6–56–13MYC::KAN pol30::URA3 leu2::YIp128-HisPOL30[LEU2]*	this study
X6386–1D	*rad5-QD::KAN pol30::URA3 leu2::YIp128-HisPOL30[LEU2]*	this study
T1646	*rad5-QD::KAN*	this study
X6141–9C	*rad5-QD::KAN smc6–56–13MYC::KAN*	this study
X6141–9D	*mms2Δ::HIS3 rad5-QD::KAN smc6–56–13MYC::KAN*	this study
X6139–1A	*esc2Δ::KAN rad5-QD::KAN*	this study
X6138–1D	*sgs1Δ::HIS3 rad5-QD::KAN*	this study
X3247–4C	*MAT*a *mph1Δ::URA3 rad5-AA*	this study
X3248–5A	*MAT*a *shu1Δ::HIS3 rad5-AA*	this study
T775	*MATα mms2Δ::URA3*	this study
X3249–9B	*MAT*a *mms2Δ::URA3 rad5-AA*	this study
X6137–1C	*rad51Δ::LEU2 rad5-QD::KAN*	this study
X6135–1B	*mph1Δ::URA3 rad5-QD::KAN*	this study
X6136–1B	*shu1Δ::HIS3 rad5-QD::KAN*	this study
X6382–2A	*mms2Δ::HIS3 rad5-QD::KAN*	this study
PJ69–4a	*MAT*a *trp1–901 leu2–3,112 ura3–52 his3–200 gal4Δ gal80Δ LYS2::GAL1-HIS3 GAL2-ADE2 met2::GAL7-lacZ*	S. Fields
PJ69–4α	*MATα trp1–901 leu2–3,112 ura3–52 his3–200 gal4Δ gal80Δ LYS2::GAL1-HIS3 GAL2-ADE2 met2::GAL7-lacZ*	S. Fields
pOAD	*pOAD*	lab collection
pXZ388	*pOAD-RAD5*	lab collection
pXZ571	*pOAD-rad5-AA*	this study
pXZ367	*pOAD-POL30*	lab collection
pXZ439	*pOAD-REV1*	lab collection
pXZ385	*pOAD-UBC13*	lab collection
pOBD	*pOBD*	lab collection
pXZ373	*pOBD-POL30*	lab collection
pXZ442	*pOBD-REV1*	lab collection
pXZ386	*pOBD-UBC13*	lab collection
p341	*pGAD424*	this study
p343	*pGAD424-POL30*	this study
p346	*pGBT9*	this study
p347	*pGBT9-RAD5*	this study
p404	*pGBT9-rad5-AA*	this study
p442	*pGBT9-rad5–1–984 a.a*.	this study
p448	*pGBT9-rad5–1–393 a.a*.	this study

### *In vivo* PCNA ubiquitination assay

Damage-induced ubiquitination of PCNA was detected as described previously (27). In brief, strains containing His_6_-PCNA were subjected to Ni-NTA affinity purification in the presence of denaturing agents. The bound fraction was eluted with loading dye and examined by western blot using antibodies against ubiquitin (P4D1) and PCNA (27).

### *In vitro* ubiquitination assays

The sources of the recombinant proteins have been described (27). All reactions were run in a buffer containing 40 mM HEPES, pH 7.4, 50 mM NaCl and 8 mM MgAc_2_. Assays for free ubiquitin chain polymerization were set up with 1 mM ATP and 100 nM each of Uba1, Ubc13, Mms2 and ubiquitin. Rad5 (WT or AA mutant) was used at 20 nM. After incubation at 30°C for the indicated times, reactions were stopped by the addition of sodium dodecyl sulphate (SDS) sample buffer and analyzed by SDS-polyacrylamide gel electrophoresis and western blotting using anti-ubiquitin antibody (P4D1). Assays for poly-ubiquitination of PCNA were set up with 1 mM ATP, 50 nM Uba1, 100 nM each of Ubc13, Mms2 and ubiquitin, 20 nM Rad5 (WT or mutant) unless otherwise noted, and 50 nM of an N-terminal Ub-PCNA fusion protein. Reactions were incubated as above and analyzed using a rabbit polyclonal antibody against PCNA. In order to probe Rad5 activity in the absence of ATP, Ubc13 was pre-charged with the ubiquitin thioester in an 8 μl reaction containing 550 nM of Uba1 and 5.5 μM each of ATP, Ubc13 and ubiquitin (WT or K63R mutant). After incubation at 30°C for 30 min, 0.25 units/ml apyrase (New England Biolabs) were added, and incubation was continued for 10 min. The reaction was then divided into two parts. To one part, Mms2, Rad5 and Ub-PCNA were added, resulting in final concentrations of 2 μM Ubc13, ubiquitin and Ub-PCNA, as well as 200 nM Uba1, Mms2 and Rad5 in a 10 μl volume, and the reaction was incubated at 30°C for 40 min. Reaction products were analyzed as described above. The other part was diluted to the same degree with reaction buffer and used for a luciferase assay (Promega) in order to confirm depletion of ATP. Remaining ATP concentrations were calculated from a standard curve. Values were determined in triplicate in order to determine averages and standard deviations.

### *In vitro* ATPase assay

DNA-dependent ATP hydrolysis was measured using a colorimetric assay kit (PiColorLock from Innova Biosciences). Reactions of 10 μl contained 20 mM Tris-HCl, pH 7.0, 20 mM KCl, 2 mM MgCl_2_, 0.5 mM ATP, 200 ng ssDNA (ΦX174 virion, New England Biolabs) where noted, 0.1 mg/ml bovine serum albumin, 0.25 mM DTT and varying concentrations of Rad5. After incubation at 30°C for 35 min, 90 μl of ATPase assay buffer were added, and reactions were stopped by addition of PiColorLock Gold reaction mix. After 30 min further incubation at room temperature, absorbance was recorded at 660 nm. A standard curve using free inorganic phosphate was generated for quantification. Values were determined in triplicate, and standard deviations were calculated.

### Other assays

Yeast two-hybrid assays, protein extraction, western blotting, DNA damage sensitivity tests, 2D gel analysis and quantification were described previously (5). For 2D gel analysis, due to the early replication of ARS305 and surrounding regions probed here, bubble replication structures were not visible and Y-shaped structures were weak at the time points shown, as replication has been completed at this locus in most cells. 2D gel tests were performed using two different spore clones for each mutant genotype. The repeat of some of these mutants is included in Supplementary Figure S1.

## RESULTS

### The Rad5 Walker B motif supports recombination-based lesion tolerance

**Figure 1.**
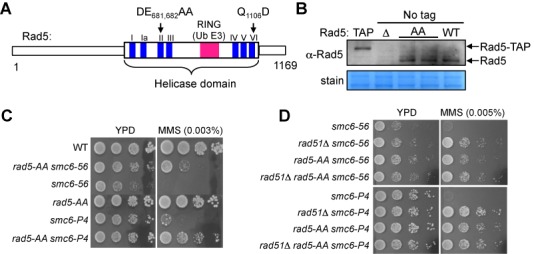
Disruption of the Rad5 Walker B motif suppresses MMS sensitivity of *smc6* mutants. **(A)** Schematic of Rad5 highlights the seven consensus motifs of the helicase domain (blue) as well as the RING ubiquitin E3 domain (pink), which is embedded in the helicase domain. Mutations in the conserved Walker B motif (DE_681, 682_AA) and conserved motif VI (Q_1106_D) are indicated. **(B)** Mutation of the Walker B motif does not affect Rad5 protein levels. Whole cell lysates from wild-type (WT) strains and strains containing TAP-tagged Rad5 (TAP), a *RAD5* deletion (Δ) and the Rad5 Walker B mutation (AA) were examined by immunoblotting using an anti-Rad5 antibody (top, Santa Cruz Biotechnology). Bands corresponding to Rad5 and TAP-tagged Rad5 are marked. Equal loading was confirmed by amido black staining (bottom). **(C)** Mutation of the Walker B motif leads to suppression of the MMS sensitivities of two *smc6* mutants (*smc6-P4* and *smc6–56*). 10-fold serial dilutions of exponentially growing cultures of the indicated strains were spotted onto normal media (YPD) and media containing the indicated concentration of MMS. **(D)***rad5-AA* and *rad51Δ* suppress the MMS sensitivities of *smc6* mutants to a similar degree and show epistatic relationships. Experiments were done as in (C).

To understand the *in vivo* function of the Rad5 helicase activity, we generated mutations at two invariable residues in the Walker B motif of the helicase domain (Figure 1A). These changes, D681A and E682A, have been shown to abolish Rad5 ATPase, helicase and fork regression activities *in vitro* (13,28). The allele with these mutations (referred to as *rad5-AA*) was integrated at the *RAD5* genomic locus without any tag to preserve regulatory elements and to avoid functional interference. Unlike Walker A motif mutations that reduce protein levels (25), *rad5-AA* sustained wild-type levels of protein (Figure 1B).

We first used a genetic readout to assess whether *rad5-AA* affects recombination-based lesion tolerance. In general, this reaction entails the formation and resolution of recombination structures. The DNA damage sensitivity caused by defective resolution of recombination structures can be rescued by reducing their formation, likely due to the mitigation of the deleterious effect of persistent recombination structures (e.g. 4,5,7,29-31). We took advantage of this feature and tested whether *rad5-AA* could rescue the DNA damage sensitivity of resolution-defective mutants, such as *smc6* alleles. We found that *rad5-AA* suppressed the sensitivity of two *smc6* mutants to the replication-stalling agent, methyl methanesulfonate (MMS) (Figure 1C). When *rad5-AA* was combined with deletion of *RAD51*, which encodes a key recombination factor, the double mutant showed a similar level of suppression as either the *rad51Δ* or the *rad5-AA* single mutant (Figure 1D). This genetic relationship suggests that the Walker B motif of Rad5 supports recombination-based processes.

### Rad5 helicase domain contributes to the Mms2-mediated recombination process

**Figure 2.**
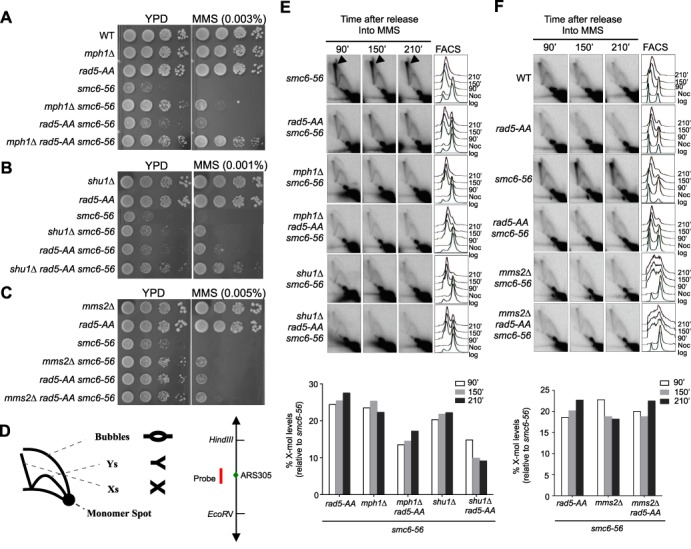
*rad5-AA* and *mms2Δ* are epistatic in their effects on *smc6*. (**A** and **B**) *mph1Δ rad5-AA* (A) or *shu1Δ rad5-AA* (B) double mutants confer greater suppression of the MMS sensitivity of *smc6–56* than the single mutants. (**C**) *rad5-AA* and *mms2Δ* confer similar suppression of *smc6–56* and are epistatic. Experiments in (A–C) were performed as in Figure 1C. (**D**) Schematics of different DNA structures detected by 2D gel and the DNA fragment analyzed. Left, DNA structures detected on 2D gel are indicated: Xs represent recombination structures; Ys and bubbles are replication intermediates. Right, an EcoRV-HindIII 3.9 kb fragment centered around the early fired origin ARS305 is analyzed in 2D gel in (E and F). The position of the probe is highlighted in red. (**E**) *rad5-AA* reduces X-mol levels in *smc6–56* cells and shows additivity with *mph1Δ* and *shu1Δ*. (**F**) *rad5-AA* reduces X-mol levels in *smc6–56* cells epistatically with *mms2Δ*. 2D gel analysis and quantification of X-mol levels (E and F) were performed as described previously (5). X-mols are indicated by arrowheads (only in the first row of E); FACS analyses are presented on the right. For simplicity, quantification of the 2D gel is shown only for *smc6–56* strains that contain deletions of recombination genes and/or *rad5-AA* (bottom).

Additional factors besides Rad51 contribute to recombination-based lesion tolerance. These include the Mms2-Ubc13 E2 enzyme that cooperates with Rad5 in PCNA poly-ubiquitination (14,15,17,29). Also, the Mph1 DNA helicase and the Rad51 paralog Shu complex act independently of each other and Mms2-Ubc13 in generating recombination structures during replication stress, as shown previously (5,30,32). To understand if *rad5-AA* affects a recombination-based mechanism mediated by these factors, we tested how *rad5-AA* relates to their mutations. Combination of *rad5-AA* with either *mph1Δ* or *shu1Δ* conferred better suppression of *smc6* MMS sensitivity than did either single mutation (Figure 2A and B). In contrast, the *rad5-AA mms2Δ* double mutant did not show better *smc6* suppression compared with either the *rad5-AA* or the *mms2Δ* single mutant, both of which suppressed *smc6* to similar levels (Figure 2C). These results suggest that *rad5-AA* and *mms2* affect a similar lesion tolerance process in an *smc6* background.

*mms2Δ* suppression of *smc6* MMS sensitivity correlates with a reduction in levels of recombination intermediate (30). In both readouts, *mms2Δ* shows additivity with *mph1Δ* and *shuΔ* (30). As *rad5-AA* is epistatic with *mms2Δ* in suppressing *smc6* MMS sensitivity, we examined whether *rad5-AA* also reduces recombination intermediate levels in *smc6* mutants and if so, how this relates to *mms2Δ, mph1Δ* and *shu1Δ*. To this end, we used agarose 2D gel analysis to visualize recombination intermediates.

Cells were synchronized in G2/M phase with nocodazole and released into the cell cycle in the presence of a sub-lethal dose of MMS. DNA was extracted at different time points and examined by 2D gel using a probe for the early-firing replication origin, ARS305 (Figure 2D). In this analysis, recombination intermediates migrate as X-shaped molecules, and are thus referred to as X-mols (4,5,7,29). We found that *rad5-AA*, like *mph1Δ, shu1Δ* and *mms2Δ*, reduced the amount of X-mols in *smc6–56* cells (Figure 2E and F; Supplementary Figure S1). As in the MMS sensitivity assay, combining *rad5-AA* with either *mph1Δ* or *shu1Δ* further decreased X-mol levels in *smc6* cells, whereas *rad5-AA mms2Δ* decreased X-mol levels to a similar degree as either *rad5-AA* or *mms2Δ* (Figure 2E and F; Supplementary Figure S1). Taken together, our results suggest that the Rad5 helicase domain promotes Mms2-mediated recombination processes.

### Intact Rad5 Walker B motif is required for PCNA poly-ubiquitination *in vivo*

**Figure 3.**
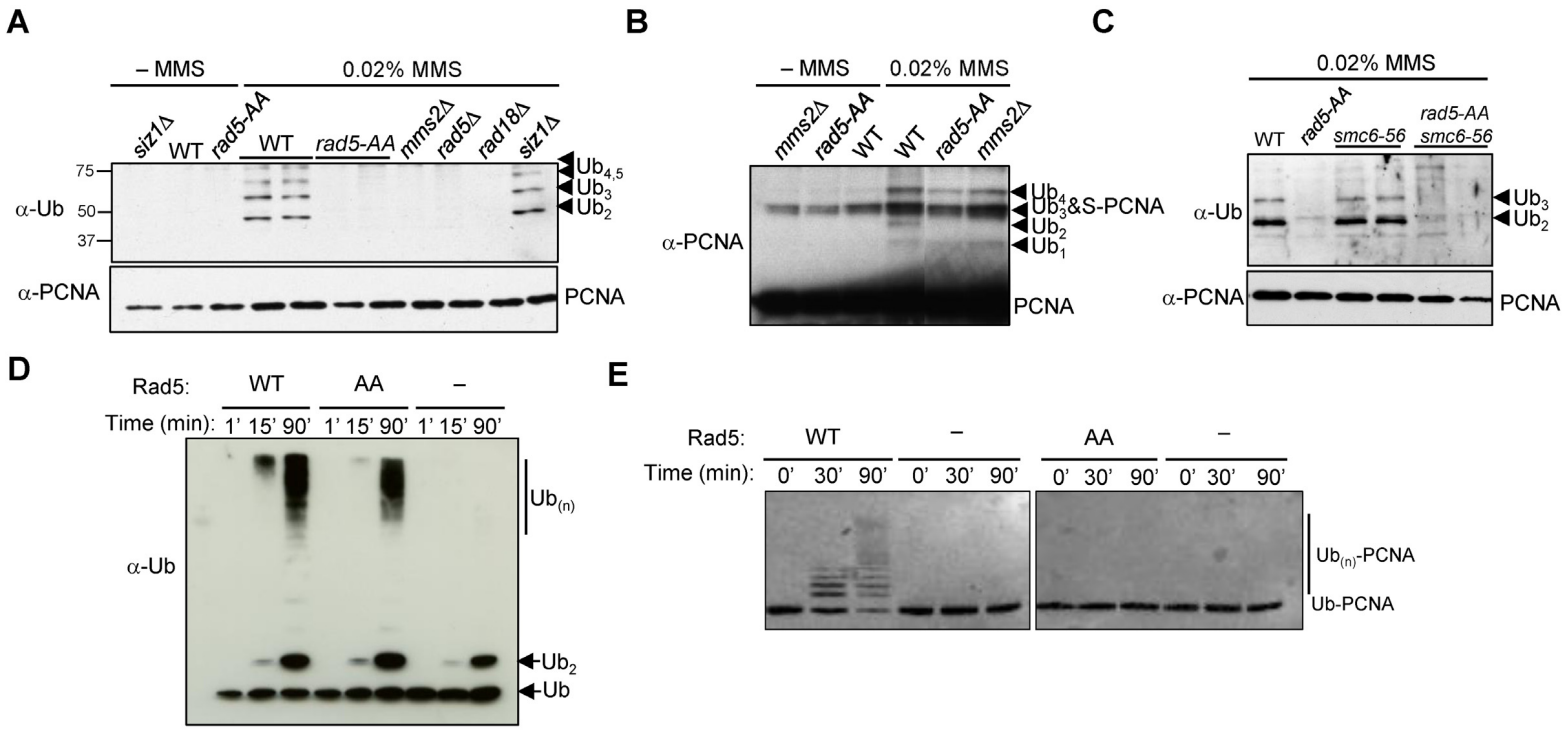
*rad5-AA* abolishes PCNA poly-ubiquitination *in vivo* and *in vitro*. (**A** and **C**) *rad5-AA* abolishes poly-ubiquitination of PCNA in both WT and *smc6* mutant backgrounds. His_6_-PCNA was purified from strains with indicated genotypes and examined by immunoblotting using anti-ubiquitin (top) or anti-PCNA (bottom) antibody. PCNA modified with two (Ub_2_) and multiple (Ub_3_, Ub_4_, Ub_5_) ubiquitin moieties was detected after treatment of WT or *smc6–56* cells with 0.02% MMS. In contrast, poly-ubiquitinated forms are absent in *rad5-AA* or deletion mutants of the PCNA ubiquitination enzymes, Mms2, Rad18 and Rad5. Removal of the SUMO-E3 Siz1, which does not affect PCNA poly-ubiquitination, was used as a control. Note that the anti-ubiquitin antibody used in this experiment does not recognize mono-ubiquitinated PCNA as reported previously (15,34). (**B**) *rad5-AA* does not affect PCNA mono-ubiquitination or sumoylation. Long exposures of the western blot using anti-PCNA antibody allow visualization of mono-ubiquitinated PCNA (Ub_1_) in addition to PCNA modified with additional ubiquitin units or with SUMO (S-PCNA). The PCNA-Ub_3_ and S-PCNA co-migrate on the gel as previously shown (15). (**D**) The rad5-AA mutant protein stimulates free ubiquitin chain formation *in vitro*. Stimulation of Ubc13-Mms2-catalyzed ubiquitin polymerization by Rad5 (WT or AA mutant) was assayed with purified proteins as previously described (17). Reaction times are indicated. (**E**) Recombinant rad5-AA protein is defective in PCNA poly-ubiquitination *in vitro*. An N-terminal fusion of ubiquitin to PCNA was used to monitor substrate-specific poly-ubiquitination by purified Rad5 (WT or AA mutant) and Mms2-Ubc13 *in vitro*, as previously described (17). Reaction times are indicated.

To understand how *rad5-AA* influences Mms2-mediated recombination tolerance, we tested whether *rad5-AA* affects PCNA poly-ubiquitination, the only known function of Mms2 in budding yeast (15,33). Using an established method, His_6_-tagged PCNA was pulled down from wild-type and mutant cell extracts, prepared before and after treatment of the cells with MMS (27). We confirmed previous findings that MMS induces PCNA poly-ubiquitination, and that the modification requires Rad18, Mms2 and Rad5, but not the PCNA sumoylation enzyme Siz1 (Figure 3A) (15,34,35). Strikingly, *rad5-AA*, like *mms2Δ*, completely abolished PCNA poly-ubiquitination but not mono-ubiquitination or sumoylation (Figure 3A and B; Supplementary Figure S2). A similar effect was also seen in the *smc6* mutant background (Figure 3C). These results provide an explanation for the genetic data and suggest that an intact helicase domain is required for PCNA poly-ubiquitination in cells. We note our results are consistent with a recent report of the absence of di-ubiquitinated PCNA in *rad5-AA siz1Δ* cells (24).

### Intact Rad5 Walker B motif is also required for PCNA poly-ubiquitination *in vitro*

The abolition of PCNA poly-ubiquitination by *rad5-AA* in cells could be due to two distinct mechanisms. First, Rad5′s ATPase or helicase activity may act upstream of PCNA modification, for example, by promoting fork reversal. Alternatively, the Walker B mutation may impair PCNA modification independently of ATPase or helicase activity. To distinguish between these possibilities, we examined *in vitro* PCNA ubiquitination with purified proteins in the absence of DNA, thus precluding the influence of ATPase or helicase activity.

First, we examined the general ubiquitin ligase activity of the rad5-AA mutant protein by monitoring the formation of unanchored poly-ubiquitin chains. Using an established assay with purified ubiquitin, E1, the Mms2-Ubc13 E2 and Rad5, we detected robust stimulation of poly-ubiquitin chain formation by Rad5 (Figure 3D). In the absence of Rad5, Mms2-Ubc13 merely promoted the formation of ubiquitin dimers under these conditions (Figure 3D) (17). The rad5-AA mutant protein was largely proficient for stimulating free ubiquitin polymerization, though a moderate reduction in its activity was observed (Figure 3D). We conclude that the rad5-AA mutant protein is proficient as a ligase for poly-ubiquitin chain formation.

To examine substrate-specific poly-ubiquitination, an N-terminal fusion of ubiquitin to PCNA (Ub-PCNA) that mimics mono-ubiquitinated PCNA was used as a substrate (17). Consistent with previous reports, Ub-PCNA is conjugated to poly-ubiquitin chains by Mms2-Ubc13 and Rad5 (Figure 3E) (17). In striking contrast to the moderate effect on free poly-ubiquitin chain synthesis, rad5-AA completely failed to poly-ubiquitinate Ub-PCNA (Figure 3E). These results show that the Rad5 Walker B mutation directly impairs PCNA poly-ubiquitination in the absence of DNA, thus independently of its DNA-dependent ATPase or helicase function.

### The Rad5 ubiquitin ligase activity does not require ATP

**Figure 4.**
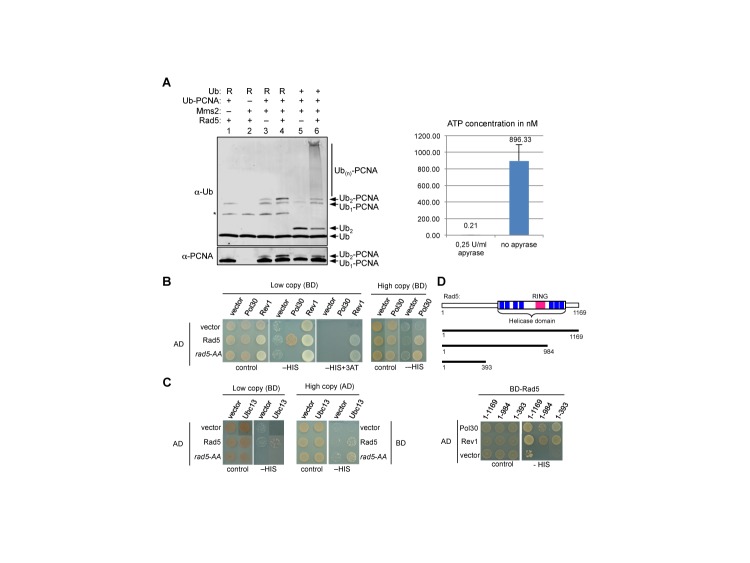
The Rad5 ubiquitin ligase function does not require ATP, and rad5-AA is defective in interactions with both PCNA and E2. (**A**) Rad5-mediated poly-ubiquitination of PCNA *in vitro* proceeds in the absence of ATP. The Ubc13∼Ub thioester was pre-formed in a reaction containing E1, Ubc13, ubiquitin and ATP. After adding apyrase for 10 min to quench ATP, the reaction was split into two samples. One part was used for a poly-ubiquitination reaction by addition of Ub-PCNA, Rad5 and Mms2 as indicated, and the reaction products were detected by Western blotting for PCNA and ubiquitin (left panel). The other part was used for a luciferase-based ATP assay in order to confirm depletion of ATP (right panel). Replacement of Ub with Ub containing the K63R mutation is indicated by ‘R’. The asterisk marks a side product of the ubiquitination reaction, the Ubc13-Ub conjugate. ATP concentrations were derived from a standard curve. (**B**) Rad5 Walker B motif contributes to PCNA interaction. Yeast two-hybrid assays (2H) using low copy plasmids (left panel) show that interaction between PCNA and Rad5, but not between Rev1 and Rad5, is abolished by the Rad5 Walker B mutation. High expression level of interactors compensates for this defect (right panel). BD: Gal4 DNA binding domain vector; AD: Gal4 activation domain. (**C**) Rad5 Walker B motif mutation affects interaction with the ubiquitin E2 Ubc13. 2H assay using low copy (left panel) but not high copy (right panel) plasmids show that the *rad5-AA* mutation abolishes the Rad5–Ubc13 interaction. (**D**) The Rad5 helicase domain is dispensable for interaction with PCNA. A schematic of Rad5 constructs used is shown at the top. The N-terminal domain of Rad5 (1–393 a.a.) as well as a larger construct (1–984 a.a.), like full length Rad5 (1–1169 a.a.), exhibits PCNA interaction in 2H assays (bottom).

The striking difference between rad5-AA's ability to promote free ubiquitin chain formation and its inability to support PCNA ubiquitination *in vitro* indicates a unique defect when PCNA is the substrate. We considered two possible explanations for this specificity. First, since the Walker B motif is required for ATP hydrolysis, this function could be particularly important for PCNA poly-ubiquitination. To test this idea, we asked whether the Rad5-mediated transfer of ubiquitin from E2 to PCNA requires ATP. In general, all ubiquitination assays contain ATP, as the E1 requires ATP for ubiquitin activation (36). In order to probe Rad5 ligase activity in the absence of ATP, the conjugation reaction was carried out in two steps. First, Ubc13 was charged with ubiquitin by E1 in the presence of ATP. In order to prevent the synthesis of unanchored polyubiquitin chains, this was done in the absence of Mms2. Apyrase was then added to quench ATP (Figure 4A, right) before transfer of the ubiquitin to PCNA was initiated by addition of Ub-PCNA, Mms2 and Rad5 (Figure 4A, left). To facilitate quantification, a ubiquitin mutant with lysine 63 replaced by arginine was tested in parallel, as the K63-specificity of the Mms2-Ubc13 dimer would limit the reaction with mutant ubiquitin to a single transfer, thus generating a distinct species of di-ubiquitinated PCNA (15,33). As expected, Rad5, Mms2 and lysine 63 of ubiquitin are required for generating poly-ubiquitinated PCNA (Figure 4A, left). Importantly, this reaction occurs in the absence of ATP. These results show that ATP hydrolysis is not required for Rad5-mediated PCNA poly-ubiquitination.

### The Walker B motif promotes Rad5 interaction with PCNA and Ubc13

After excluding a requirement of ATP hydrolysis for Rad5′s ubiquitin ligase function, we examined whether rad5-AA is defective in bridging PCNA and E2, a reported function of Rad5 (14,15,17). Using the yeast two-hybrid assay (2H), we found that Rad5 and rad5-AA showed similar interactions with a known interactor, Rev1 (Figure 4B, left) (37,38), suggesting that *rad5-AA* does not affect general protein folding. When expressed from low-copy-number plasmids (CEN-based), Rad5, but not rad5-AA, showed interaction with PCNA and Ubc13 (Figure 4B and C, left), indicating that the rad5-AA mutant is defective in associations with substrate and E2. We note that Ball *et al*. report a lack of interaction between rad5-AA and Ubc13 but did not examine PCNA interaction (24). Interestingly, our observations indicate a reduction rather than an abolition of interaction, as increasing protein levels by using high-copy-number plasmids (2μ-based) restored interactions with PCNA (Figure 4B, right) and Ubc13 (Figure 4C, right). As rad5-AA supports free ubiquitin chain formation (Figure 3D), reduced interaction with Ubc13 appears to be tolerated in this aspect of the ligase function. In contrast, simultaneous reductions in interactions with both E2 and substrate likely account for the abolition of the ligase activity on PCNA.

The above 2H results prompted us to examine whether the Walker B-containing region in Rad5 serves as the PCNA-binding domain. Using a series of deletion constructs of Rad5, we found that the first 393 a.a. of the protein are sufficient for PCNA association (Figure 4D). This is consistent with a previous inference based on *in vitro* ubiquitination assays (14). This region overlaps with the Rev1-binding region (37), suggesting that the Rad5 N-terminal serves as a scaffold for multiple protein-protein interactions (Figure 4D). We conclude that the Walker B motif facilitates but is unlikely directly involved in the PCNA-Rad5 interaction.

### A distinct *rad5* helicase mutation sustains PCNA modification

**Figure 5.**
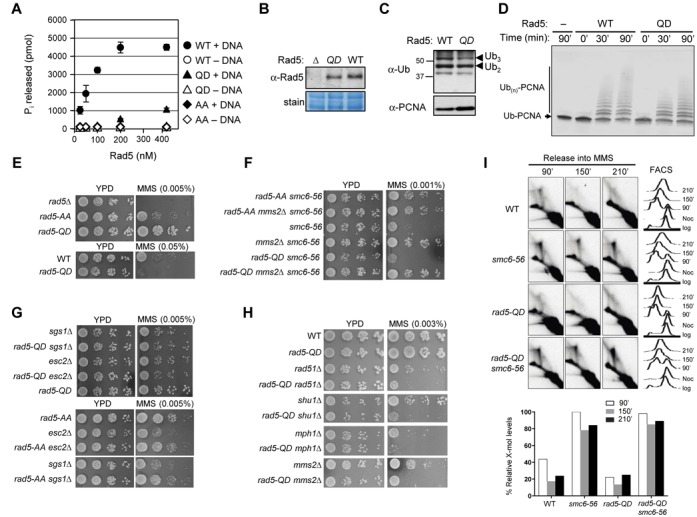
Rad5 helicase activity is dispensable for PCNA poly-ubiquitination and *smc6* suppression. (**A**) The rad5-QD mutant protein exhibits a severe defect in DNA-dependent ATP hydrolysis *in vitro*. ATPase activity was monitored by measuring the amount of inorganic phosphate released as a function of protein concentration. Note that no activity was detected for all three proteins in the absence of DNA. (**B**) *rad5-QD* mutation does not affect the Rad5 protein level. Experiment was done as in Figure 1B. (**C**) *rad5-QD* is proficient for PCNA poly-ubiquitination *in vivo*. Experiment was done as in Figure 3A. (**D**) The rad5-QD mutant protein supports poly-ubiquitination of PCNA *in vitro*. Experiment was done as in Figure 3E, but using 200 nM each of Mms2, Ubc13 and Rad5, 50 nM Uba1, 10 μM ubiquitin and 2 μM Ub-PCNA. (**E**) *rad5-QD* is less sensitive to MMS than *rad5-AA* and *rad5Δ* mutant strains. (**F**) *rad5-QD*, unlike *rad5-AA*, does not suppress the MMS sensitivity of *smc6–56*. (**G**) *rad5-QD*, unlike *rad5-AA*, does not suppress the MMS sensitivity of *sgs1Δ* and *esc2Δ* cells. (**H**) *rad5-QD* sensitizes mutants impaired in replication-associated recombination. Mutations of recombination factors and of proteins with non-overlapping roles in replication-associated recombination, namely Mms2, Shu1 and Mph1, were combined with *rad5-QD*. Experiments in (E–H) were performed as in Figure 1C. (**I**) *rad5-QD* does not reduce the X-mol levels in *smc6–56* cells. The genomic DNA of the indicated strains was digested with NcoI and the replication/recombination intermediates forming in the proximity of the early origin of replication ARS305 were visualized with the ARS305 probe. Experiments were done as in Figure 2E and F.

Our results suggest that the Walker B motif of Rad5 contributes to PCNA poly-ubiquitination by promoting substrate-enzyme interaction. This premise disfavors, but does not exclude, an *in vivo* requirement of Rad5 ATPase or helicase activity for PCNA poly-ubiquitination. To test this possibility directly, we constructed another Rad5 helicase mutation by replacing the invariant Q1106 in motif VI of the helicase domain (referred to as *rad5-QD*). Studies of multiple helicases similar to Rad5 have shown that motif VI and the invariant Q within it are required for ATPase activity; thus, mutation of this residue is often used as a bona fide helicase mutation (e.g. 39,40). Examination of the purified mutant protein in an *in vitro* ATPase assay indeed confirmed a severe defect in DNA-dependent ATP hydrolysis (Figure 5A).

We also verified by western blotting that *rad5-QD* did not affect protein levels in cells (Figure 5B). In striking contrast to *rad5-AA, rad5-QD* mutant cells exhibited similar levels of PCNA poly-ubiquitination as wild-type control cells (Figures 3A and B and 5C). Consistent with this observation, the rad5-QD protein was fully competent in supporting poly-ubiquitination of Ub-PCNA (Figure 5D) and substrate-independent ubiquitin polymerization *in vitro* (Supplementary Figure S3). These results indicate that the Rad5 ATPase or helicase activity *per se* is not required for PCNA modification *in vivo* or *in vitro*. In conjunction with our *rad5-AA* results, this finding supports the notion that *rad5-AA* is a doubly defective allele, simultaneously deficient in PCNA poly-ubiquitination and in ATPase or helicase activity, whereas *rad5-QD* is a true separation-of-function allele that affects only the latter. In line with this conclusion, *rad5-AA* cells were more MMS sensitive than *rad5-QD* cells, which showed sensitivity only at high MMS doses (Figure 5E).

### The Rad5 ATPase activity is dispensable for recombination-based lesion tolerance

Next, we used *rad5-QD* to address whether the sole lack of the helicase activity of Rad5 affects recombination-based lesion tolerance. Using a similar readout as before (Figure 1), we found that unlike *rad5-AA, mms2Δ* or their double mutant, *rad5-QD* did not affect the MMS sensitivity of *smc6* mutants (Figures 1C, 2C and 5F). In addition, *rad5-QD* had no effect on the suppression of *smc6* conferred by *mms2Δ* (Figure 5F). Thus, Rad5 ATPase activity does not affect *smc6* survival in the presence or absence of Mms2.

Besides Smc5/6, the dissolution enzyme subunit Sgs1 and the scaffold protein Esc2 are also required for the resolution of recombination structures generated during replication (6,7,30,31,41). While *rad5-QD* showed no effect on the MMS sensitivity of mutants lacking Sgs1 and Esc2, *rad5-AA* exhibited suppression (Figure 5G). Moreover, when we combined *rad5-QD* with *rad51Δ, mph1Δ, shu1Δ* and *mms2Δ*, the double mutants of *rad5-QD* and each of these deletions were more sensitive to MMS than the corresponding single mutants (Figure 5H). Finally, different from *rad5-AA* and mutants of other factors that contribute to damage-bypass via recombination (Figure 2E and F), *rad5-QD* did not markedly reduce the level of X-mols (Figure 5I). These results together strongly support the notion that Rad5′s ATPase or helicase activity promotes recombination-independent lesion tolerance and is functionally separable from PCNA ubiquitination.

## DISCUSSION

Many important proteins involved in DNA metabolism possess two or more activities. The multi-functionality of these proteins suggests a central role in genome maintenance, but also hinders efforts to delineate how they act in their different capacities. In this study, we examined one such protein, the yeast Rad5 DNA helicase and ubiquitin ligase. Rad5 and its homologs have similar structural organizations, with the ubiquitin ligase domain embedded within the larger helicase domain (reviewed in 23). A similar organization is found in several other DNA repair proteins, such as Rad16, Uls1 and Irc20 (reviewed in 23). In all cases, it is unclear how the very different activities within the same protein influence and are differentiated from each other. In the case of Rad5, previous analyses of a number of mutations in the helicase motifs led to different conclusions regarding the effect of the protein's helicase activity on its ubiquitin ligase activity (e.g. 9,11,24,25). In the two most recent studies, Ortiz-Bazan *et al*. concluded that the two activities work sequentially and not independently, whereas Ball *et al*. suggested both possibilities (11,24). This disagreement prevents the generation of a unifying picture of how Rad5 functions *in vivo*. To address the controversy in the field, we examined a Walker B mutation and an additional, unrelated helicase mutation using genetic, biochemical and 2D gel analyses. Our findings uncover a new mechanism by which the helicase domain supports the Rad5 ubiquitin ligase activity on a specific substrate by fostering substrate-enzyme interaction, and this function is in addition to its known role in ATP hydrolysis. While the Walker B motif is required for both activities, we establish that only the E3 ligase activity leads to recombination-based lesion tolerance, whereas the ATPase activity makes separate contributions under replication stress conditions. These findings suggest both overlapping and distinct biological effects of the two Rad5 activity domains.

### Rad5 Walker B motif supports PCNA poly-ubiquitination and a branch of lesion bypass

We show that *rad5-AA* by itself and in resolution-defective mutants specifically abolishes multiple forms of PCNA poly-ubiquitination, but not sumoylation (Figure 3A–C; Supplementary Figure S2). These results extend a recent finding of the absence of di-ubiquitinated PCNA in *rad5-AA siz1Δ* cells (24). Importantly, our 2D gel data provide the first direct evidence that *rad5-AA* impairs recombination-based lesion bypass (Figure 2E and F). These findings corroborate our genetic analyses of *rad5-AA* with mutations in factors involved in the formation (Rad51, Mph1, Shu and Mms2) and resolution (Smc6, Sgs1 and Esc2) of recombination intermediates (Figures 1C and D and 2A–C). By providing both physical and genetic evidence for *rad5-AA* influence on lesion bypass, our data delineate the specific effect of *rad5-AA* on a branch of recombination-based lesion bypass. These findings extend the genetic analyses using fewer mutants (24), and reveal the biological consequences of mutating the Rad5 Walker B motif in multiple resolution-defective mutants.

### Mechanism by which the Rad5 helicase domain supports PCNA poly-ubiquitination

We define the mechanisms by which the Walker B motif affects PCNA poly-ubiquitination using several assays. We show that rad5-AA is proficient for free poly-ubiquitin chain formation, but completely failed to ubiquitinate PCNA *in vitro* (Figure 3D and E). As this defect is evident in the absence of DNA, any influence of a potential helicase activity can be excluded. Consistent with this, we show that wild-type Rad5 catalyzes PCNA poly-ubiquitination in an ATP-independent manner (Figure 4A). These results demonstrate that Walker B motif does not affect PCNA poly-ubiquitination by influencing the general ubiquitin ligase activity, ATP hydrolysis or DNA helicase activity. These findings, in conjunction with the analyses of *rad5-QD* (see below), repudiate the postulation that Rad5 helicase function is required for PCNA poly-ubiquitination (11,24).

Using yeast two-hybrid analysis, we show that rad5-AA impairs but does not abolish interactions with both Ubc13 and PCNA (Figure 4B and C). The slight reduction in rad5-AA's ability to promote free ubiquitin chain synthesis (Figure 3D) is in line with a partial reduction in Ubc13 binding (Figure 4C), and possibly recruitment as suggested previously (24). However, the complete loss of ligase activity towards PCNA can be explained only by the simultaneous reductions in rad5-AA interactions with Ubc13 and PCNA, rather than the sole loss of Ubc13 interaction, a conclusion different from Ball *et al*. (24). Our data additionally indicate that the N-terminal region of Rad5 is sufficient for interaction with PCNA (Figure 4D). Based on these findings and the known mechanisms of other E3s, we propose that the helicase domain is involved in positioning the embedded RING finger in a productive conformation for ubiquitin transfer from the E2 to the substrate, rather than directly binding to either. Future structural studies will provide a detailed mechanistic understanding, illuminating how the helicase domain plays a structural role. It will be interesting to understand whether the new function of the Rad5 helicase domain uncovered here reflects a general principle among proteins with analogous domain arrangements.

### Rad5 ATPase activity promotes replication stress tolerance independently of recombination

Our data suggest that *rad5-QD* is the first genuine separation-of-function allele that does not affect PCNA poly-ubiquitination or protein levels (Figure 5A–D). Consistent with this notion, *rad5-QD* cells are less sensitive to MMS than *rad5-AA* cells (Figure 5E). The use of *rad5-QD* allows us to assess how lack of Rad5 helicase function alone affects recombination-based lesion bypass. We show that Rad5 ATPase activity *per se* supports lesion survival independently of recombination. Unlike *mms2Δ* or *rad5-AA, rad5-QD* does not alter the damage sensitivity of resolution-defective mutants (Figure 5F and G). In addition, *rad5-QD* does not reduce the levels of recombination intermediates in *smc6* mutants (Figure 5I). Furthermore, *rad5-QD* exhibits additive relationships with pro-recombination mutants (Figure 5H). This last finding shows that although Rad5 helicase activity *per se* may not be a strong determinant of MMS resistance, it becomes critical when recombination is impaired. As *rad5-AA* has been used as a helicase defective-only mutation in some studies prior to the knowledge of its effect on PCNA modification (e.g. 9,13,28), reexamination using *rad5-QD* will help to clarify the effect of helicase function in genome maintenance functions. We also note that the phenotype of *rad5-QD* contrasts with that caused by the lack of Mph1, the only other known yeast DNA helicase that can catalyze fork regression *in vitro* (5,13,30,31,42). This difference raises the possibility that the helicase catalyzing fork reversal may be able to dictate different outcomes following this reaction.

**Figure 6.**
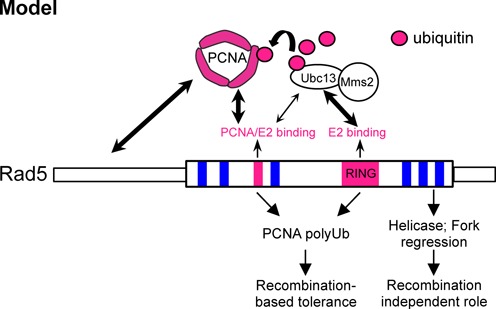
Model summarizing our findings. See text for details.

The model in Figure 6 highlights our finding that Rad5-mediated PCNA poly-ubiquitination and Rad5 ATPase activity make separable contributions to overcoming replication stress. Consistent with previous findings, the Rad5 ligase activity, through PCNA poly-ubiquitination, promotes recombination-based lesion tolerance. Our findings suggest that its ATPase activity apparently plays a recombination-independent role. On the other hand, the helicase domain, in combination with the N-terminal and ligase domains, contributes to PCNA ubiquitination by supporting enzyme-substrate interaction. Since the *rad5-AA* mutant is not as sensitive to DNA damage as *rad5Δ*, Rad5 clearly has other functions, such as in translesion synthesis or double-strand break repair (25,28,37,38,43,44). Further work is needed to elucidate the *in vivo* replication and repair situations under which each of these activities can take effect.

## SUPPLEMENTARY DATA

Supplementary Data are available at NAR Online.

SUPPLEMENTARY DATA

## References

[1] Branzei D. (2011). Ubiquitin family modifications and template switching. FEBS Lett..

[2] Chang D.J., Cimprich K.A. (2009). DNA damage tolerance: when it's OK to make mistakes. Nat. Chem. Biol..

[3] Ulrich H.D. (2014). Two-way communications between ubiquitin-like modifiers and DNA. Nat. Struct. Mol. Biol..

[4] Branzei D., Sollier J., Liberi G., Zhao X., Maeda D., Seki M., Enomoto T., Ohta K., Foiani M. (2006). Ubc9- and mms21-mediated sumoylation counteracts recombinogenic events at damaged replication forks. Cell.

[5] Chen Y.-H., Choi K., Szakal B., Arenz J., Duan X., Ye H., Branzei D., Zhao X. (2009). Interplay between the Smc5/6 complex and the Mph1 helicase in recombinational repair. Proc. Natl Acad. Sci. U.S.A..

[6] Liberi G., Maffioletti G., Lucca C., Chiolo I., Baryshnikova A., Cotta-Ramusino C., Lopes M., Pellicioli A., Haber J.E., Foiani M. (2005). Rad51-dependent DNA structures accumulate at damaged replication forks in sgs1 mutants defective in the yeast ortholog of BLM RecQ helicase. Genes Dev..

[7] Sollier J., Driscoll R., Castellucci F., Foiani M., Jackson S.P., Branzei D. (2009). The Saccharomyces cerevisiae Esc2 and Smc5–6 proteins promote sister chromatid junction-mediated intra-S repair. Mol. Biol. Cell.

[8] Johnson R.E., Henderson S.T., Petes T.D., Prakash S., Bankmann M., Prakash L. (1992). Saccharomyces cerevisiae RAD5-encoded DNA repair protein contains DNA helicase and zinc-binding sequence motifs and affects the stability of simple repetitive sequences in the genome. Mol. Cell. Biol..

[9] Minca E.C., Kowalski D. (2010). Multiple Rad5 activities mediate sister chromatid recombination to bypass DNA damage at stalled replication forks. Mol. Cell.

[10] Moinova H.R., Chen W.-D., Shen L., Smiraglia D., Olechnowicz J., Ravi L., Kasturi L., Myeroff L., Plass C., Parsons R. (2002). HLTF gene silencing in human colon cancer. Proc. Natl Acad. Sci. U.S.A..

[11] Ortiz-Bazán M.Á., Gallo-Fernández M., Saugar I., Jiménez-Martín A., Vázquez M.V., Tercero J.A. (2014). Rad5 plays a major role in the cellular response to DNA damage during chromosome replication. Cell. Rep..

[12] Sood R., Makalowska I., Galdzicki M., Hu P., Eddings E., Robbins C.M., Moses T., Namkoong J., Chen S., Trent J.M. (2003). Cloning and characterization of a novel gene, SHPRH, encoding a conserved putative protein with SNF2/helicase and PHD-finger domains from the 6q24 region. Genomics.

[13] Blastyák A., Pintér L., Unk I., Prakash L., Prakash S., Haracska L. (2007). Yeast Rad5 protein required for postreplication repair has a DNA helicase activity specific for replication fork regression. Mol. Cell.

[14] Carlile C.M., Pickart C.M., Matunis M.J., Cohen R.E. (2009). Synthesis of free and proliferating cell nuclear antigen-bound polyubiquitin chains by the RING E3 ubiquitin ligase Rad5. J. Biol. Chem..

[15] Hoege C., Pfander B., Moldovan G.-L., Pyrowolakis G., Jentsch S. (2002). RAD6-dependent DNA repair is linked to modification of PCNA by ubiquitin and SUMO. Nature.

[16] Johnson R.E., Prakash S., Prakash L. (1994). Yeast DNA repair protein RAD5 that promotes instability of simple repetitive sequences is a DNA-dependent ATPase. J. Biol. Chem..

[17] Parker J.L., Ulrich H.D. (2009). Mechanistic analysis of PCNA poly-ubiquitylation by the ubiquitin protein ligases Rad18 and Rad5. EMBO J..

[18] Atkinson J., McGlynn P. (2009). Replication fork reversal and the maintenance of genome stability. Nucleic Acids Res..

[19] Carr A.M., Lambert S. (2013). Replication stress-induced genome instability: the dark side of replication maintenance by homologous recombination. J. Mol. Biol..

[20] Eisen J.A., Sweder K.S., Hanawalt P.C. (1995). Evolution of the SNF2 family of proteins: subfamilies with distinct sequences and functions. Nucleic Acids Res..

[21] Ulrich H.D. (2003). Protein-protein interactions within an E2-RING finger complex. Implications for ubiquitin-dependent DNA damage repair. J. Biol. Chem..

[22] Ulrich H.D., Jentsch S. (2000). Two RING finger proteins mediate cooperation between ubiquitin-conjugating enzymes in DNA repair. EMBO J..

[23] Unk I., Hajdú I., Blastyák A., Haracska L. (2010). Role of yeast Rad5 and its human orthologs, HLTF and SHPRH in DNA damage tolerance. DNA Repair.

[24] Ball L.G., Xu X., Blackwell S., Hanna M.D., Lambrecht A.D., Xiao W. (2014). The Rad5 helicase activity is dispensable for error-free DNA post-replication repair. DNA Repair.

[25] Chen S., Davies A.A., Sagan D., Ulrich H.D. (2005). The RING finger ATPase Rad5p of Saccharomyces cerevisiae contributes to DNA double-strand break repair in a ubiquitin-independent manner. Nucleic Acids Res..

[26] Zhao X., Blobel G. (2005). A SUMO ligase is part of a nuclear multiprotein complex that affects DNA repair and chromosomal organization. Proc. Natl Acad. Sci. U.S.A..

[27] Ulrich H.D., Davies A.A. (2009). Methods in Molecular Biology.

[28] Gangavarapu V., Haracska L., Unk I., Johnson R.E., Prakash S., Prakash L. (2006). Mms2-Ubc13-dependent and -independent roles of Rad5 ubiquitin ligase in postreplication repair and translesion DNA synthesis in Saccharomyces cerevisiae. Mol. Cell. Biol..

[29] Branzei D., Vanoli F., Foiani M. (2008). SUMOylation regulates Rad18-mediated template switch. Nature.

[30] Choi K., Szakal B., Chen Y.-H., Branzei D., Zhao X. (2010). The Smc5/6 complex and Esc2 influence multiple replication-associated recombination processes in Saccharomyces cerevisiae. Mol. Biol. Cell.

[31] Mankouri H.W., Ngo H.-P., Hickson I.D. (2009). Esc2 and Sgs1 act in functionally distinct branches of the homologous recombination repair pathway in Saccharomyces cerevisiae. Mol. Biol. Cell.

[32] Chavez A., Agrawal V., Johnson F.B. (2011). Homologous recombination-dependent rescue of deficiency in the structural maintenance of chromosomes (Smc) 5/6 complex. J. Biol. Chem..

[33] Hofmann R.M., Pickart C.M. (1999). Noncanonical MMS2-encoded ubiquitin-conjugating enzyme functions in assembly of novel polyubiquitin chains for DNA repair. Cell.

[34] Papouli E., Chen S., Davies A.A., Huttner D., Krejci L., Sung P., Ulrich H.D. (2005). Crosstalk between SUMO and ubiquitin on PCNA is mediated by recruitment of the helicase Srs2p. Mol. Cell.

[35] Windecker H., Ulrich H.D. (2008). Architecture and assembly of poly-SUMO chains on PCNA in Saccharomyces cerevisiae. J. Mol. Biol..

[36] Haas A.L., Warms J.V., Hershko A., Rose I.A. (1982). Ubiquitin-activating enzyme. Mechanism and role in protein-ubiquitin conjugation. J. Biol. Chem..

[37] Kuang L., Kou H., Xie Z., Zhou Y., Feng X., Wang L., Wang Z. (2013). A non-catalytic function of Rev1 in translesion DNA synthesis and mutagenesis is mediated by its stable interaction with Rad5. DNA Repair.

[38] Pagès V., Bresson A., Acharya N., Prakash S., Fuchs R.P., Prakash L. (2008). Requirement of Rad5 for DNA polymerase zeta-dependent translesion synthesis in Saccharomyces cerevisiae. Genetics.

[39] Gross C.H., Shuman S. (1996). The QRxGRxGRxxxG motif of the vaccinia virus DExH box RNA helicase NPH-II is required for ATP hydrolysis and RNA unwinding but not for RNA binding. J. Virol..

[40] Pause A., Sonenberg N. (1992). Mutational analysis of a DEAD box RNA helicase: the mammalian translation initiation factor eIF-4A. EMBO J..

[41] Mankouri H.W., Ngo H.-P., Hickson I.D. (2007). Shu proteins promote the formation of homologous recombination intermediates that are processed by Sgs1-Rmi1-Top3. Mol. Biol. Cell.

[42] Zheng X.F., Prakash R., Saro D., Longerich S., Niu H., Sung P. (2011). Processing of DNA structures via DNA unwinding and branch migration by the S. cerevisiae Mph1 protein. DNA Repair.

[43] Acharya N., Johnson R.E., Prakash S., Prakash L. (2006). Complex formation with Rev1 enhances the proficiency of Saccharomyces cerevisiae DNA polymerase zeta for mismatch extension and for extension opposite from DNA lesions. Mol. Cell. Biol..

[44] Pages V., Santa Maria S.R., Prakash L., Prakash S. (2009). Role of DNA damage-induced replication checkpoint in promoting lesion bypass by translesion synthesis in yeast. Genes Dev..

