# Supplementary material for: Concerted and differential actions of two enzymatic domains underlie Rad5 contributions to DNA damage tolerance
Source: Nucleic Acids Res. 2015 Feb 17;43(5):2666–77. doi: 10.1093/nar/gkv004 (PMC4357696; doi:10.1093/nar/gkv004)
Supplement: SUPPLEMENTARY DATA [file supp_43_5_2666__index.html]

Concerted and differential actions of two enzymatic domains underlie Rad5 contributions to DNA damage tolerance — SUPPLEMENTARY DATA 

# Concerted and differential actions of two enzymatic domains underlie Rad5 contributions to DNA damage tolerance

## SUPPLEMENTARY DATA

**Files in this Data Supplement:**

- Supplementary Figures
