# Supplementary material for: Concerted and differential actions of two enzymatic domains underlie Rad5 contributions to DNA damage tolerance
Source: Nucleic Acids Res. 2015 Feb 17;43(5):2666–77. doi: 10.1093/nar/gkv004 (PMC4357696; doi:10.1093/nar/gkv004)
Supplement: SUPPLEMENTARY DATA [file supp_gkv004_nar-03142-h-2014-File008.pdf]

Supplementary Fig 1

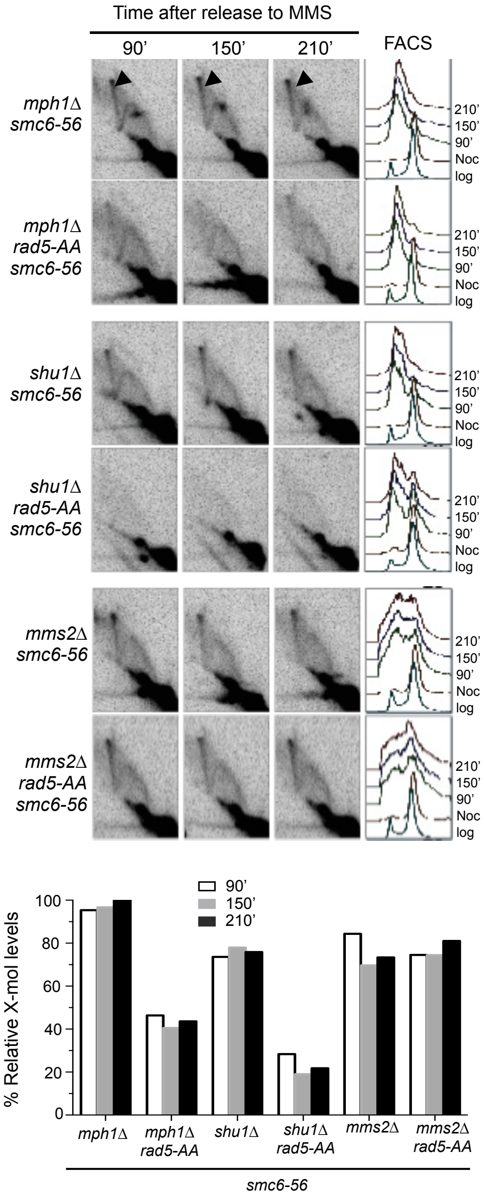

Supplementary Figure 1. 2D gel examination of recombination intermediates of additional spore isolates to confirm the results in Figure 2E and 2F. Experiments were performed as those in Figures 2E–2F, except different spore isolates of the indicated genotypes were examined. Consistent with Figures 2E–2F, in *smc6-56* background, *rad5-AA* further reduces the levels of recombination intermediates (arrow heads) when combined with *mph1*Δ or *shu1*Δ, but it is largely epistatic with *mms2*Δ.

Supplementary Fig 2

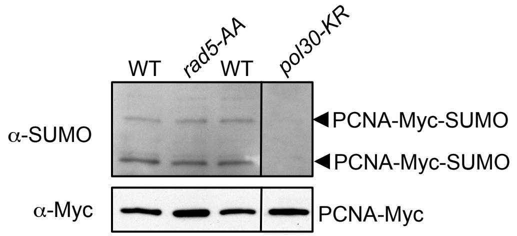

Supplementary Figure 2. *rad5-AA* exhibits wild-type level of PCNA sumoylation. Myc-tagged PCNA was immuno-precipitated and examined by anti-Myc and anti-SUMO antibodies. The absence of PCNA sumoylated forms is evident in mutant PCNA with mutated sumoylation sites (Pol30-KR) as shown previously.

Supplementary Fig 3

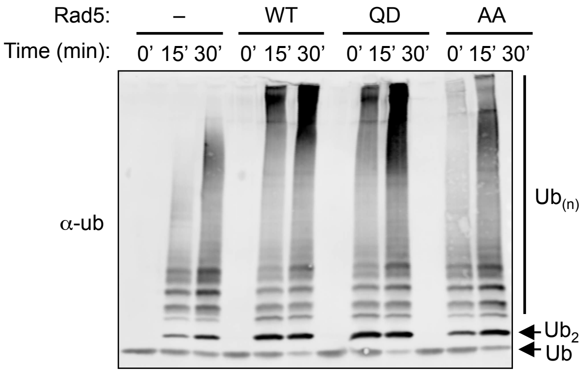

Supplementary Figure 3. The rad5-QD mutant stimulates free ubiquitin chain formation *in vitro*. The experiment was performed as in Figure 3D, but using 200 nM each of Mms2, Ubc13 and Rad5, 50 nM Uba1 (E1) and 10 mM ubiquitin.
